# Genus-Wide Pan-Genome Analysis of *Oryza* Calcium-Dependent Protein Kinase Genes and Their Related Kinases Highlights the Complexity of Protein Domain Architectures and Expression Dynamics

**DOI:** 10.3390/plants14101542

**Published:** 2025-05-20

**Authors:** Fu Shi, Li Li, Mingjie Chen, Junli Chang, Min Tu, Guangyuan He, Yin Li, Guangxiao Yang

**Affiliations:** 1 The Genetic Engineering International Cooperation Base of Chinese Ministry of Science and Technology, The Key Laboratory of Molecular Biophysics of Chinese Ministry of Education, College of Life Science and Technology, Huazhong University of Science & Technology, Wuhan 430074, China2024511001@hust.edu.cn (L.L.); cmj@hust.edu.cn (M.C.); cjl@hust.edu.cn (J.C.); 2 Hubei Province Key Laboratory of Agricultural Waste Resource Utilization, Hubei Technical Engineering Research Center for Chemical Utilization and Engineering Development of Agricultural and Byproduct Resources, School of Chemical and Environmental Engineering, Wuhan Polytechnic University, Wuhan 430023, China

**Keywords:** calcium-dependent protein kinases, CPKs and their related kinases, *Oryza*, duplicated gene, EF-hand motifs, transcriptome analysis

## Abstract

The *Oryza* genus serves not only as a gene pool for rice improvement but also as a model system for plant evolutionary research. Calcium-dependent protein kinases (CPKs) function as both effectors and sensors in calcium signaling and play versatile roles in plant development and stress responses. Four kinase families, namely CPK-related kinases (CRKs), phosphoenolpyruvate carboxylase kinases (PPCKs), PPCK-related kinases (PEPRKs), and calcium- and calmodulin-dependent kinases (CCaMKs), are frequently called CPK-related kinases. This study utilized evolutionary genomics approaches and provided the pan-genome repertoires of *CPKs* and their related kinases in 34 *Oryza* genomes by leveraging the rich genomics resources of the *Orzya* genus. Gene duplication analysis revealed that distinct duplication types contributed to expanding *CPKs* and their related kinases in wild rice. We depicted the protein domain architectures of CPKs and their related kinases, highlighting the complexity of EF-hand motifs in CPKs and CCaMKs. Transcriptome analysis determined that alternative splicing was a mechanism contributing to the diversity in the domain architectures of CPKs and CCaMKs. We also generated the expression atlas of *CPKs* and their related kinases in multiple species of *Oryza* genus, emphasizing divergent homoeolog expression patterns across tissues and species in allotetraploid wild rice. Collectively, our *Oryza*-wide analysis of *CPKs* and their related kinases revealed their evolutionary trajectories and highlighted their diversified domain architectures and expression dynamics, providing gene resources of wild relatives for rice improvement.

## 1. Introduction

Rice is the most widely consumed staple crop, feeding over half of the world’s population [1]. However, rice production is facing enormous challenges: the increasing demands arising from a growing global population and the escalating threats to rice yield posed by climate changes. To address these challenges, increasing yield and environmental adaptability is urgent for rice improvement. The species of *Oryza* genus has been considered an important gene pool for cultivated rice and contains a vast array of genetic diversity that can be harnessed to enhance various traits in cultivated rice, such as disease resistance, stress tolerance, and yield potential [2].

The *Oryza* genus belongs to the grass family (*Poaceae*) and encompasses 2 cultivated species (Asian cultivated rice (*Oryza sativa*) and African cultivated rice (*Oryza glaberrima*)) and 25 wild relatives, including 11 distinct genome types (AA, BB, CC, EE, FF, GG, BBCC, CCDD, HHJJ, HHKK, and KKLL) [3]. Additionally, these wild relatives exhibit significant phenotypic diversity. On one hand, wild relatives have numerous desirable traits enabling their great adaptability to different ecosystems, owing to their diverse genotypes and extensive geographical distribution. For example, *Oryza longistaminata* possesses special traits that give it strong resistance to biotic and abiotic stresses and rhizomatousness [4]. On the other hand, some favorable genes were lost during rice domestication and/or the improvement of rice, and the genetic diversity of modern rice varieties has been reduced by human selection during rice improvement and breeding processes. Thus, mining favorable genes from wild relatives and re-identifying lost genes or alleles from wild relatives serve as promising strategies for rice improvement.

Recently, high-quality genome assemblies of the species of *Oryza* genus have been available for comparative and functional genomics studies due to the advances in genomics technologies. To date, more than 68 genome assemblies of wild rice species have been published, covering almost all genome types in *Oryza*, except for HHJJ and HHKK [4,5,6,7,8,9,10]. For cultivated rice, based on the 3000 Rice Genomes Project [11], Asian cultivated rice has been classified into nine subpopulations. Subsequently, more than 40 high-quality genomes [12,13,14], representing subpopulations of Asian cultivated rice, have been de novo assembled. These genomic resources allow us to mine favorable genes and investigate the evolutionary relationships of gene families in *Oryza*. Importantly, the *Oryza* genus has diversified genotypes and phenotypes within a narrow evolutionary time scale (~15 million years) with several closely spaced speciation events, recording a nearly stepwise evolutionary history [15]. *O. sativa* and *O. glaberrima* were independently domesticated from *Oryza rufipogon* and *Oryza barthii*, respectively [16]. Recently, de-domestication has been revealed in weedy rice (*Oryza sativa f. spontanea*), which are mostly derived from local cultivated rice [17]. The *Oryza* genus has roles in evolutionary dynamics, domestication, de-domestication, and speciation, making it a suitable genus model for evolutionary and comparative genomics studies.

In parallel to the advances in monocot genome assemblies (such as those in the *Oryza* genus), evolutionary genomics has unraveled several ancestral whole genome duplication (WGD) events, important for genetic innovation and expanding genetic contents [18,19,20,21]. In particular, lineage-specific features of gene duplication have been identified with potential contributions to the speciation and improvement of crop species at both the whole genome and gene family levels, such as in the Triticeae tribe [22,23]. In line with this concept, a new comparative genomics strategy has been proposed. This strategy emphasizes integrating gene duplication and genome-wide analyses (iGG) to facilitate cross-species gene identification and the reverse-genetics discovery of functional genes [23]. Although these new resources and strategies have been established for crop genomics, their utilization has been scarce in *Oryza*, an agriculturally important genus with probably the richest genomics resources.

This study aimed to leverage the rich genomics resources and to exemplify the utilization of the iGG strategy in the *Oryza* genus. As such, we chose calcium-dependent protein kinases (CPKs) and their related kinases to provide comprehensive bioinformatic characterization, because they represent a set of protein kinase families crucial for plant development and environmental adaptability. We believe that this study will serve as a starting point for utilizing evolutionary genomics-based strategies to identify gene members valuable for rice improvement. To rapidly respond to environment stimulus and coordinate environmental conditions and growth, plants have evolved several protein kinase families to facilitate calcium signaling and cellular signaling transduction. Among these kinases, CPKs and the four families, namely CPK-related kinases (CRKs), phosphoenolpyruvate carboxylase kinases (PPCKs), PPCK-related kinases (PEPRKs), and calcium- and calmodulin-dependent kinases (CCaMKs), are functionally important. CPKs serve as both sensors and effectors of Ca^2+^ signaling, while the other four related families are evolutionarily close to CPKs with differentiated structures, thus being frequently referred as CPK-related kinases [24,25,26,27]. Hereafter, we refer to CPKs and these above-mentioned kinases as CPKs and their related kinases.

In terms of distribution across taxa, *CPKs* are found in plants, as well as in green algae and protozoa (ciliates and apicomplexans), whereas *CRKs*, *CCaMKs*, *PPCKs*, and *PEPRKs* are plant-specific, despite the absence of *CCaMK* in *Arabidopsis* [24]. In rice, at least twenty-nine *OsCPKs*, five *OsCRKs*, two *OsPEPRKs*, three *OsPPCKs*, and one *OsCCaMK* have been identified [28,29,30]. Functionally, CPKs play versatile roles in plant development, stress tolerance, and plant immunity [31,32,33]. Unlike CPKs, relatively fewer functions have been reported for CRKs in plants, revealing their roles in root growth and hormonal responses [34,35]. CCaMKs function as regulators in root nodules and Arbuscular mycorrhizal symbioses in legumes [36]. OsCCaMK/OsDMI3 is reported to be involved in the response to abiotic and biotic stresses [37]. PPCKs phosphorylate PEP carboxylase, a key enzyme in C4 and CAM pathways. *Arabidopsis AtPPCKs* are reported to respond to phosphate and salt stresses [38]. Regarding PEPRKs, expression analyses suggest that they might be transcriptionally regulated in response to abiotic stresses and phytohormones.

Structurally, CPKs and their related kinases contain distinct regulatory domains. CPKs consist of four domains, namely a variable N-terminal domain (VNTD), a kinase domain, an autoinhibitory junction domain (JD), and a C-terminal calmodulin (CaM)-like domain (CaMLD) with four EF-hand motifs. Most CPKs contain potential myristoylation and palmitoylation sites in the VNTD, and these sites are associated with subcellular localization. Ca^2+^ could bind EF-hand motifs and regulate the activity of CPKs. CRKs share similar structures with CPKs but lack functional EF-hand motifs in their CaMLDs, making them unable to bind to Ca^2+^. Some CRKs are reported to interact with CaM for regulation. PPCKs are characterized by a single kinase domain, while regulatory domains are absent in the C-terminals. PEPRKs possess a kinase domain and a distinct C-terminal domain. Their kinase domains are closely related to PPCKs, while their C-terminal domains differ from the remaining CPKs and their related kinases. In contrast, CCaMKs contain a kinase domain, an overlapping autoinhibitory region/CaM-binding domain, and a visinin-like Ca^2+^-binding domain. Due to the unique structures, CCaMKs are regulated through both Ca^2+^ and CaM [24,26]. Because of the significance of Ca^2+^ signaling in the plant response to environmental cues and the critical roles of CPKs and their related kinases in environmental adaptability and coordination between growth and environmental conditions, we speculated that the gene copy numbers and/or protein structures of CPKs and their related kinases might exhibit dynamic variation within the *Oryza* genus, especially between the cultivated rice species and their wild relatives.

Here, we provided the first pan-genome repertoire of *CPKs* and their related kinases in the 34 *Oryza* genomes. Gene duplication analysis revealed that different types of duplication events contributed to the expansion of *CPKs* and their related kinases in wild rice. We depicted protein domain architectures of CPKs and their related kinases, highlighting the complexity of EF-hand motifs in *Oryza* CPKs and CCaMKs. Transcriptome analysis found that alternative splicing contributed to the diversity in the protein domain architectures of CPKs and CCaMKs. We also generated the expression atlas of *CPKs* and their related kinases in multiple species of *Oryza* genus. Our homoeolog expression analysis uncovered divergent homoeolog expression bias patterns between species and tissues in allotetraploid wild rice. This updated knowledge of *CPKs* and their related kinases deepens our understanding of the regulation mechanisms of CPKs and their related kinases and provides gene resources from wild relatives for rice improvement.

## 2. Results

### 2.1. The Pan-Genome Analyses Enabled Understanding the Repertoire of CPKs and Their Related Kinases in Oryza

**Figure 1:**
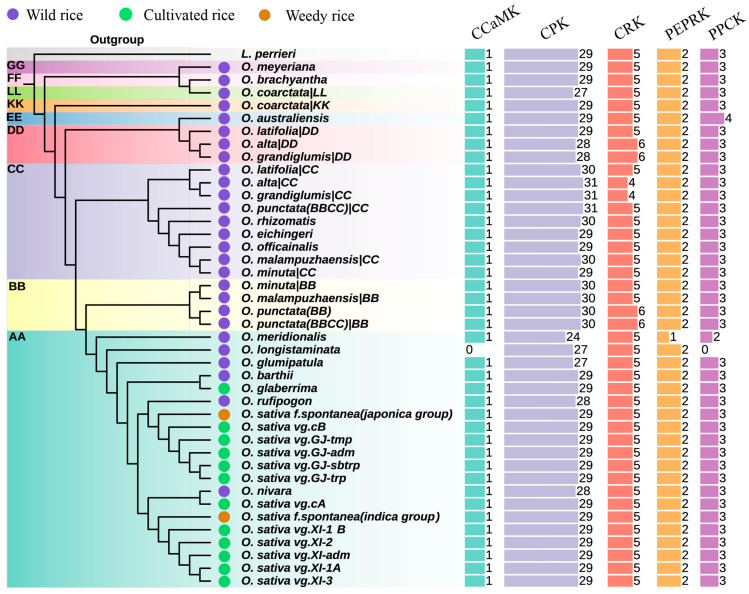
Gene numbers of *CPKs* and their related kinases in *Oryza*. The identified gene numbers of *CPKs* and their related kinases in 34 *Oryza* genomes and the *L. perrieri* outgroup. Please note that the subgenomes of allotetraploid rice have been separated and treated as a diploid genome in this figure. The colored bars represent the gene numbers of the *CCaMK*, *CPK*, *CRK*, *PEPRK*, and *PPCK* gene families. The evolutionary tree was constructed based on 2297 orthologous genes, with *L. perrieri* being the outgroup. Wild, cultivated, and weedy rice are indicated in the tree with purple, green, and brown circles, respectively. Corresponding genome/subgenome types (AA, BB, CC, DD, EE, KK, LL, FF, and GG) are labeled in the evolutionary tree.

To obtain a comprehensive *Oryza* catalog of the genes encoding CPKs and their related kinases, we selected 34 representative genome assemblies in *Oryza* covering nine genome types (AA, BB, CC, EE, FF, GG, BBCC, CCDD, and KKLL), including 20 wild rice, 12 cultivated rice, and 2 weedy rice accessions, with *Leersia perrieri* being the outgroup (Figure 1 and Table S1). Firstly, we systematically re-identified these gene families in three publicly available Nipponbare reference annotations (MUS [39], AGIS-1.0 [40], and RAP-DB [41]) via Bitacora [42], an innovative pipeline integrating sequence similarity-based search algorithms (BLAST and HMMER) and a homology-based gene prediction program GeMoMa v1.9 [43], allowing us to scan genomic sequences to precisely identify gene family members not curated in genome annotation. A total of 40 genes, comprising 1 *CCaMK*, 29 *CPKs*, 5 *CRKs*, 2 *PEPRKs*, and 3 *PPCKs*, were identified in each annotation without newly identified genes (Table S2). We further characterized the gene structures of the encoding genes and protein domain architectures of OsCPKs and their related kinases (Figure S1). Considering the accuracy and completeness of annotations, our identified CPKs and their related kinases from the Nipponbare MUS annotation and *Arabidopsis thaliana* Col-0 (TAIR10) were used as the seeds for the gene identification of CPKs and their related kinases in the *Oryza* genus.

We systematically identified 1676 genes encoding CPKs and their related kinases, including 41 *CCaMKs*, 1217 *CPKs*, 212 *CRKs*, 83 *PEPPKs*, and 123 *PPCKs*, in *Oryza* (Figure 1 and Table S3). Notably, the cultivated rice and weedy rice accessions shared the same gene numbers of *CPKs* and their related kinases, while some wild rice accessions had more kinase-encoding genes than those in the cultivated rice (e.g., *PPCKs* in *Oryza australiensis*, *CRKs* in *Oryza alta* and *Oryza punctata* (BB), and *CPKs* in *Oryza malampuzhaensis*), underlining species-specific expansions of certain kinase families in the *Oryza* genus (Figure 1). Our pan-genome catalogs of *CPKs* and their related kinases in *Oryza* laid the foundation for their evolutionary relationships, protein domain architectures, and expression analysis.

**Figure 2:**
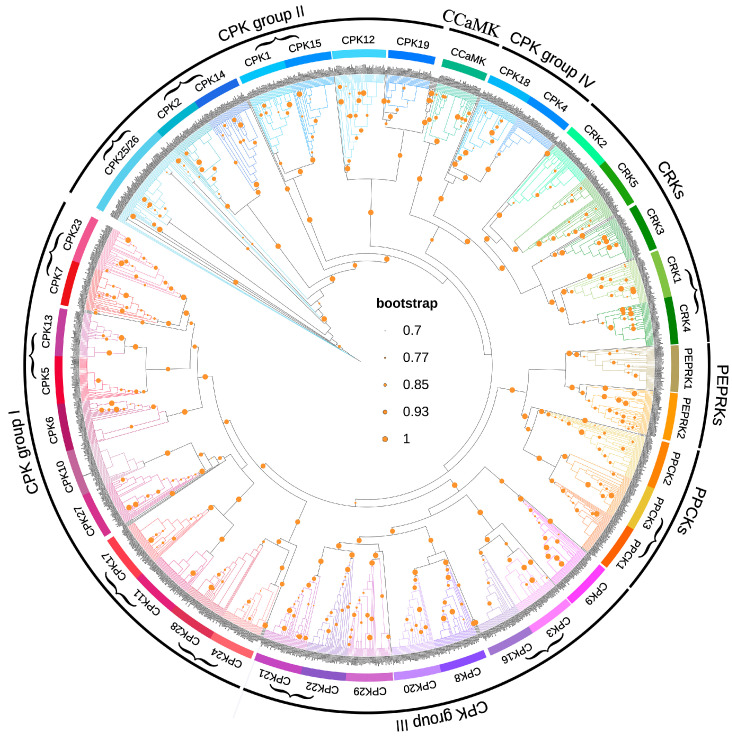
A phylogenetic analysis of the CPKs and their related kinases. The identified CPKs and their related kinases from wild, cultivated, and weedy rice, along with their members in *Arabidopsis*, were used in the phylogenetic analysis. The 1676 identified CPKs and their related kinases were cladded into eight groups, namely CPK subgroups I-IV, CRKs, PPCKs, PEPPKs, and CCaMK. Bootstrap values > 0.7 are indicated as orange circles. Duplicated gene pairs shared in *Oryza* genomes are labeled with brackets.

To understand the evolutionary relationships of CPKs and their related kinases in *Oryza*, we performed phylogenetic analysis using the protein sequences of the identified CPKs and their related kinases, together with thirty-four AtCPKs, eight AtCRKs, two AtPPCKs, and two AtPEPRKs [24]. CPKs were clustered into four subgroups, designated as CPK subgroups I-IV, while CRKs, PPCKs, PEPPKs, and CCaMKs were separately clustered, designated as CRKs, PPCKs, PEPPKs, and CCaMKs, respectively. Furthermore, CPKs from subgroup IV were closer to CRKs and CCaMKs than to CPKs from other subgroups, suggesting the more recent divergence of CRKs, at least in *Oryza* (Figure 2). In our analysis, we identified 11 duplicated gene pairs that commonly occurred in these species of *Oryza* genus (e.g., *CPK11*/*CPK17* in CPK subgroup I, *CPK1*/*CPK15* in CPK subgroup II, *CPK3*/*CPK16* in CPK subgroup III, *PPCK1*/*PPCK3*, and *CRK1*/*CRK4*), implying that these gene pairs had been duplicated before *Oryza* speciation, while no gene pair was found in CPK subgroup IV (Figure 2). Our phylogenetic analysis established the orthologous relationships of CPKs and their related kinases among these species of *Oryza* genus, facilitating a detailed analysis of gene duplication and copy number variations for genes encoding these kinases.

### 2.2. Several Types of Gene Duplication Contribute to the Expansion of CPKs and Their Related Kinases

**Figure 3:**
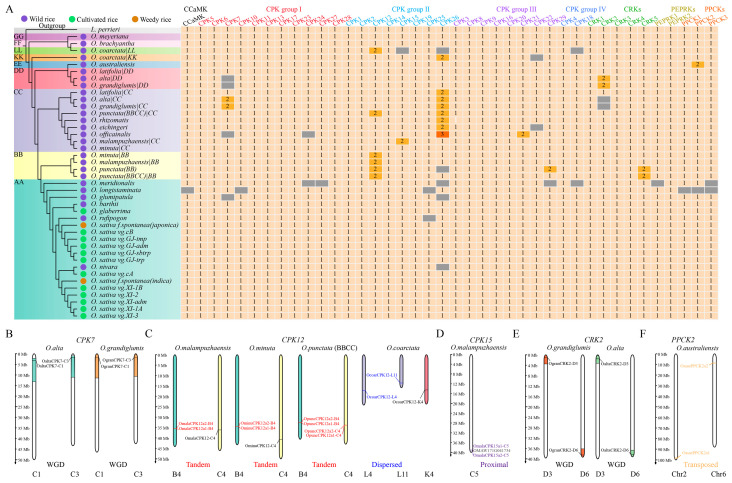
Gene duplication contributes to the expansion of *CPKs* and their related kinases in *Oryza*. (**A**) The copy numbers of genes encoding CPKs and their related kinases in genomes/subgenomes are indicated in the boxes. Gray indicates that genes are absent in corresponding genomes/subgenomes. A coalescent-based evolutionary tree was constructed based on 2297 single-copy orthologous genes with *L. perrieri* as the outgroup. Wild, cultivated, and weedy rice are indicated in tree with purple, green, and brown circles, respectively. Corresponding genome/subgenome types (AA, BB, CC, DD, EE, KK, LL, FF, and GG) are labeled in the evolutionary tree. Gene names in different colors are ordered by their evolutionary groups, namely CCaMK, CPK group I-IV, CRKs, PEPRKs, and PPCKs. (**B**–**F**) The visualization of representative gene duplication examples that contributed to the expansion of *CPK7* (**B**), *CPK12* (**C**), *CPK15* (**D**), *CRK2* (**E**), and *PPCK2* (**F**). WGD: whole genome duplication; tandem, tandem duplication; dispersed, dispersed duplication; proximal, proximal duplication; transposed, transposed duplication.

To establish the syntenic orthologous/paralogous relationships of the *Oryza* CPKs and their related kinases, we constructed collinear graphs at the inter- and intra-genome/subgenome levels. Regarding the copy number variation (CNV) in *CPKs* and their relatives in genomes/subgenomes (i.e., AA, BB, CC, DD, EE, FF, GG, KK, and LL), cultivated rice and weedy rice accessions shared the same gene numbers for each of the kinase families (one *CCaMK*, twenty-nine *CPKs*, five *CRKs*, two *PEPPKs*, and three *PPCKs*), implying that these kinase families might be fixed in cultivated rice and weedy rice (Figure 3 and Table S3). In contrast, wild rice exhibited lineage- or species-specific expansion patterns. *CCaMK* was present in single-copy form in all genomes/subgenomes, excluding its absence in *O. longistaminata* (Figure S2), which may have been due to the genome assembly quality. *CPK12s* were expanded in subgenomes BB and LL (Figure S3), and *PPCK2* was expanded in *Oryza australiensis* (EE), showing lineage-specific patterns. *CPK15* and *CPK21* were uniquely expanded in *O. malampuzhaensis* and *Oryza officainalis*, while *PEPPK1* was exclusively absent in *Oryza meridionalis*. Notably, *CPK7* was absent in the subgenome CC of *O. alta* and *Oryza grandiglumis*, while it was preserved as two copies in the subgenome DD of *O. alta* and *O. grandiglumis*, resulting in unaltered total gene numbers. A similar phenomenon was also found in *CRK2* (Figure 3 and Table S3).

Whole genome duplication (WGD), tandem duplication (TD), proximal duplication (PD), transposed duplication (TRD), and dispersed duplication (DSD) drive the expansion of gene families in plant genomes, contributing to the diversification of gene contents, structures, and functions; complex molecular network constructions; and phenotypic innovations [39]. To explore the drivers behind the expansion of *CPKs* and their related kinases in the wild rice species, we analyzed the gene duplication modes of duplicated genes, and these above-mentioned drivers contributed jointly to expanding *CPKs* and their related kinases. For instance, WGD-derived *CRK2*, *CPK7*, and *CPK21* generated two copies in *O. alta*, *O. grandiglumis*, and *O. officainalis*, respectively (Figure 3). In contrast, TRD contributed to expanding *CPK29* in *O. punctata* (BB) and *PPCK2* in *O. australiensis*, while PD drove *CPK15* expansion in *O. malampuzhaensis* (Figure 3). We also observed that the expansions of *CRK5s* in *O. punctata* (BB) and *O. punctata* (BBCC) were influenced by both WGD and TRD.

Furthermore, we identified gene duplication modes that were conservative or divergent in a genome- or subgenome-specific way. TD contributed to the expansion of *CPK12s* in the subgenome BB, whereas DSD drove *CPK12* expansion in the subgenome LL, suggesting subgenome-specific evolutionary trajectories with independent contributions to gene expansion (Figure 3). *CPK26s* gave rise to two copies with genome-specific mechanisms. TRD was the dominant driver in the subgenome CC, except for *Oryza rhizomatis* and *O. officainalis*. DSD mediated *CPK26* expansion in *O. rhizomatis*, while both WGD and PD contributed to *CPK26* expansion in *O. officainalis*. Taken together, our analyses revealed conservative and divergent gene duplication mechanisms in *Oryza*, establishing a framework for exploring how duplication modes influence the protein domain architectures and expression patterns of CPKs and their related kinases.

### 2.3. Myristoylation and Palmitoylation of CPKs and Their Related Kinases

Myristoylation and palmitoylation sites in the N-termini of CPKs and CRKs play critical roles in membrane association and biological functions, and these sites could be predicted with established bioinformatic tools [44,45]. Our analysis results showed that neither myristoylation nor palmitoylation sites were detected in all CCaMKs. Moreover, no myristoylation or palmitoylation sites were predicted in most PPCKs and PEPRKs. In contrast, myristoylation and/or palmitoylation sites were predicted in most CRKs and CPKs, except CPK24 and CPK28 (Table S3). The predicted subcellular localizations of CPKs and their related kinases also supported their myristoylation and palmitoylation sites. CPK24, CPK28, and CCaMKs without myristoylation or palmitoylation sites were predicted to be localized in cytoplasm or nucleus, while those proteins containing myristoylation and/or palmitoylation sites were predicted to target the cell membrane. The information for myristoylation and palmitoylation and predicted subcellular localization suggest differences in membrane targeting and potential functional divergence among CPKs and their related kinases.

### 2.4. Molecular Properties of CPKs and Their Related Kinases

We predicted the molecular properties (i.e., protein length, molecular weight, and isoelectric point (pI)) of 1676 identified CPKs and related kinases (Table S3). These proteins varied in length, ranging from 119 amino acids (AAs) to 1246 AAs, with their molecular weights ranging from 12.6 kDa to 140.3 kDa. These proteins also had a wide range of pI, varying from 4.39 to 9.79. CPKs from group IV displayed the narrowest length distribution (428–609 AAs), recording molecular weights between 48.5 and 68.2 kDa. In contrast, PEPRKs had the largest variations in protein length (119–1172 AAs) and molecular weight (12.6–127.3 kDa). These data demonstrated the diversity among subgroups of CPKs and their related kinases.

Gene duplication might lead to functional divergence by shaping protein domain architectures, which could be revealed via molecular characteristics. To explore the effects of gene duplication on these proteins, we compared molecular properties between duplicated gene pairs. Most duplicated gene pairs shared similar protein characteristics. For example, WGD-driven CPK7 in *O. alta* had the same protein length (547 AAs), a similar molecular weight (62.0 kDa vs. 61.9 kDa), and the same pI (5.5). However, we found changed properties of duplicated gene pairs in some cases, for example, TRD-derived *CPK26* in *Oryza eichingeri*.

### 2.5. Landscapes of Domain Architectures Reveal the Diversity of EF-Hand Motifs in CPKs and CCaMKs

**Figure 4:**
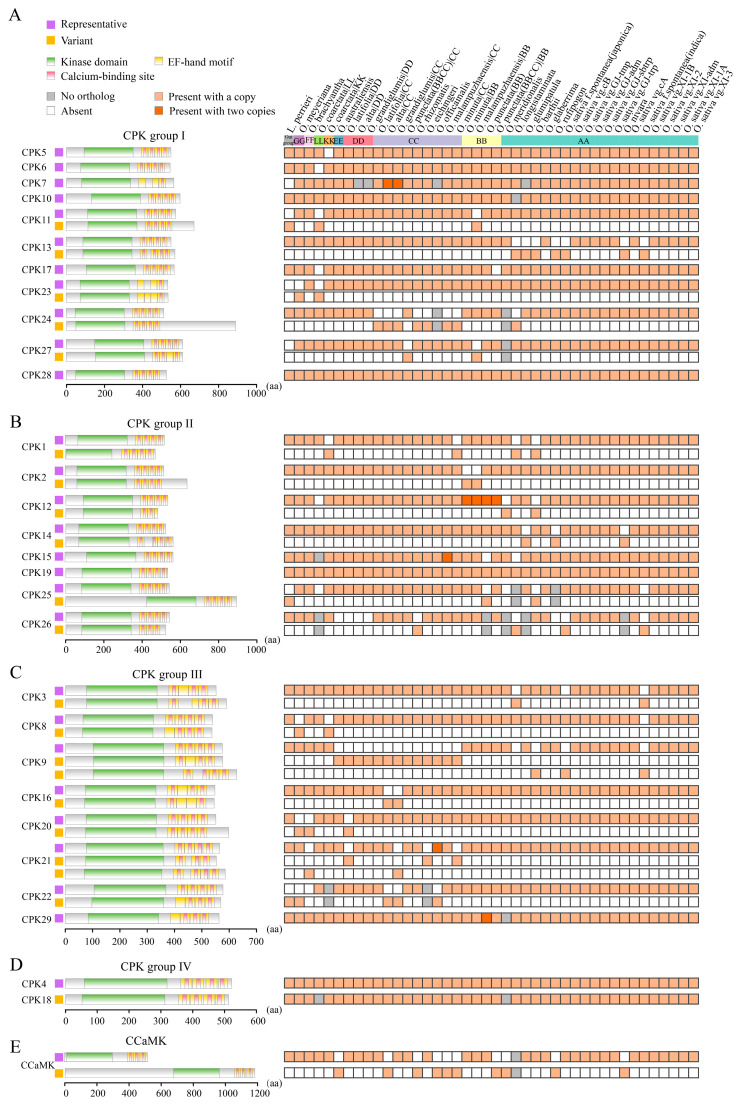
Comparative analysis of EF-hand motifs of CPKs and CCaMKs in *Oryza*. (**A**–**E**) Predicted kinase domains, EF-hand motifs, and calcium-binding sites of CPKs and CCaMKs are represented with green, yellow, and red rectangles, respectively. CPKs and CCaMKs are ordered based on evolutionary subgroups, including CPK groups I-IV (**A**–**D**) and CCaMKs (**E**). Protein domain architectures are clustered into two types: those supported by the majority of CPKs or CCaMKs were considered as the representative types (purple squares), and those supported by the minor members were regarded as variants (orange squares). The presence and absence of protein domain architecture types in corresponding genomes/subgenomes are indicated on the right squares in orange and white, respectively.

We analyzed the protein domains in the CPKs and related kinases, depicted the landscape of domain architectures, and revealed complexity in domain organization. CPKs and CCaMKs consisted of a kinase domain and variable EF-hand motifs, while PEPRKs and PPCKs only possessed a kinase domain. Compared with three EF-hand motifs in all CCaMKs, the number of EF-hand motifs in *Oryza* CPKs varied from three to four. Notably, CRK4 harbored a kinase domain and a single degenerated EF-hand motif that could not bind Ca^2^⁺, which is consistent with AtCRK3 lacking Ca^2^⁺-binding capacity despite containing two analogous EF-hand motifs [46]. The remaining CRKs only contained a kinase domain, resembling PEPRKs and PPCKs (Figure 4 and Figure S4).

According to the previous studies and our analysis, the major differences in domain architectures of CPKs and their related kinases lie in their C-terminal domains, especially in the numbers and variants of EF-hand motifs [24,26]. To address whether EF-hand motifs and Ca^2^⁺ binding sites of CPKs and CCaMKs had become divergent in *Oryza*, we compared the organizations of EF-hand motifs with a focus on Ca^2^⁺ binding sites based on identified CPKs and CCaMKs. We excluded the solo types per CPK or CCaMK and clustered CPKs and CCaMKs, respectively, according to the organization of EF-hand motifs and Ca^2^⁺ binding sites. Protein domain architectures supported by most CPKs or CCaMKs were considered the representative types, while those supported by minor CPKs or CCaMKs were regarded as the variants. By comparing the protein domain architectures, we found that most canonical CPKs generally contained four EF-hand motifs, while the duplicated gene pair CPK7/CPK23 contained three EF-hand motifs (Figure 4). In some canonical CPKs, some EF-hand motifs lacked functional Ca^2^⁺-binding sites. Notably, CPK7/CPK23 (the gene pair in group I) held three EF-hand motifs, but only with a single Ca^2^⁺-binding site in the C-lobe of the CaMLD domain. CPK3/CPK16 (the gene pair in group III) and CPK29 possessed four EF-hand motifs, with a degenerated Ca^2^⁺-binding site in the N-lobe of the CaMLD domain. Owing to the differences in Ca^2^⁺ affinity between the N-lobe and C-lobe EF-hand motifs, the altered number of functional EF-hand motifs could be associated with distinct kinase activity levels in response to basal and elevated calcium concentrations, contributing to decode calcium signals with distinct dynamics.

Comparing EF-hand motifs between variants and the corresponding representative types, some CPKs within group I (CPK5, CPK6, CPK10, CPK17, and CPK28), group II (CPK15 and CPK19), and group III (CPK29), and all group IV CPK members (CPK4 and CPK18), exhibited conservative organization of EF-hand motifs (Figure 4). Compared with the corresponding representative domain architectures, CPK12, CPK21, and CPK26 contained fewer EF-hand motifs and Ca^2+^-binding sites, while CPK8, CPK9, CPK22, and CPK27 had more Ca^2+^-binding sites, with their numbers of EF-hand motifs being unaltered. In some cases, CPKs within group I (CPK11 and CPK24), group II (CPK2), and group III (CPK20) had longer amino acid sequences after the EF-hand motifs in the C-terminus. Additionally, CPK3, CPK13, CPK14, and CPK21 contained longer interspaced sequences between the first and second EF-hand motifs (Figure 4). The divergence in EF-hand motifs and the proximal sequences could contribute to the distinct CPK-involved regulatory mechanisms.

We further explored the impacts of gene duplication on the protein domain architectures. *CPK12* experienced diverse gene duplication events, and the domain architectures of the proteins encoded by the *CPK12* duplicates diverged. The duplicates *OcoarCPK12-L4*/*OcoarCPK12-L11* were generated from dispersed duplication, leading to alterations in the protein domain architectures. *CPK12* was tandemly duplicated into two copies in *O. malampuzhaensis*, *Oryza minuta*, *O. punctata* (BB), and *O. punctata* (BBCC), respectively, while the proteins encoded by these duplicated genes exhibited divergent domain architectures (Figure S4). Similarly, the proteins encoded by the transposon-mediated duplicates *OaustPPCK2a1*/*OaustPPCK2a2* diverged in the domain architecture, with a C-terminal truncation detected in OaustPPCK2a2 (Figure S5). Conversely, the two copies of WGD-derived *CPK7* in *O. alta* and *O. grandiglumis*, respectively, both retained identical protein domain architectures (Figure S6). Like *CPK7*, *CPK15* in *O. malampuzhaensis* underwent proximal duplication, yet it also maintained the same domain structure (Figure S7). Collectively, different types of gene duplication in *Oryza* CPKs could be associated with similar or diversified domain architectures of the corresponding encoding proteins. More importantly, we depicted the landscapes of domain architectures of CPKs and CCaMKs, highlighted the complexity of EF-hand motifs and Ca^2+^-binding sites, and documented gene duplication events and their associated diversification of CPK domain architectures.

### 2.6. Alternative Splicing Contributes to the Diversity of Domain Architectures of CPKs and CCaMKs

Alternative splicing (AS) is a pivotal mechanism in transcriptional regulation and allows for producing different protein variants from the same gene loci, substantially expanding protein diversity. To explore the impact of AS in shaping protein domain architectures, we collected and processed 427 RNA-seq libraries to systematically identify the transcripts of CPKs and CCaMKs (Table S4). Protein domain architectures encoded by identified transcripts were predicted via the homology-based annotation method. With the RNA-seq-based transcript assemblies, we corrected the annotations of some genes, e.g., *OaltaCPK26a1-C12* (Figure S8). Our assembled transcript of *OaltaCPK26a1-C12.2* had a more similar gene model and protein length to the orthologous gene *OsCPK26* than the original gene (*OaltaCPK26a1-C12.1*).

**Figure 5:**
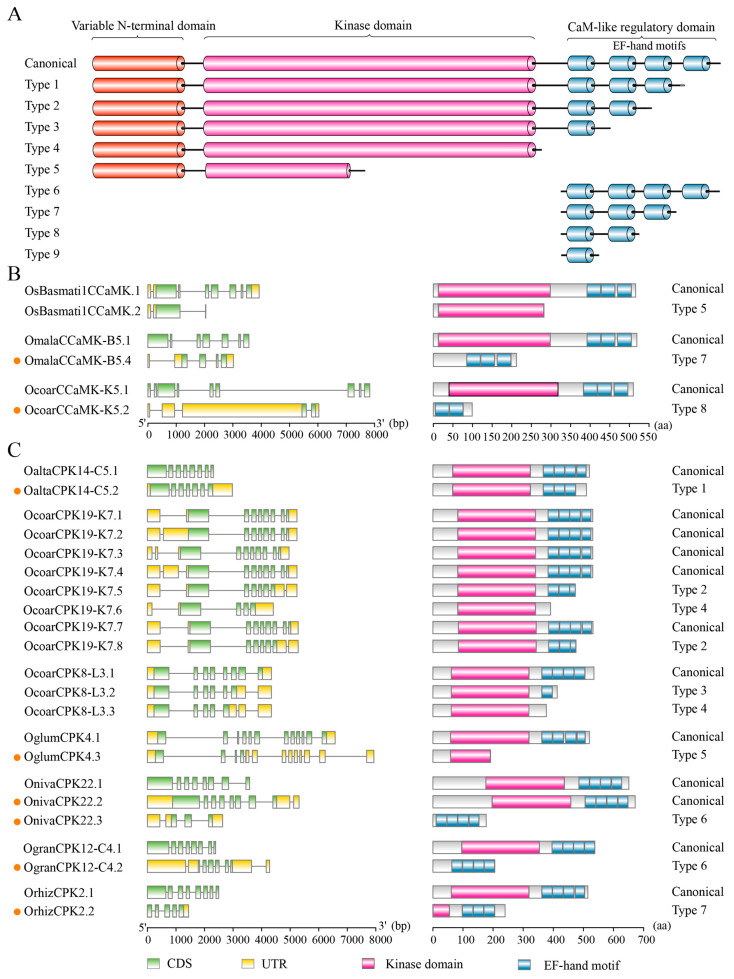
Alternative splicing contributes to the protein domain architectures of CPKs and CCaMKs. (**A**) A graphic illustration representing the nine potential domain architecture types of canonical and truncated CPKs, namely truncated EF-hand motifs (Types 1–4), truncated kinase domain (Type 5), and the remaining EF-hand motifs (Types 6–9). Variable N-terminal domains, the kinase domains, and the EF-hand motifs are shown with range, red, and blue cylinders, respectively. (**B**,**C**) Gene structures of representative transcripts of *CCaMKs* (**B**) and *CPKs* (**C**) and their encoded protein domain architectures. The reconstructed transcripts are highlighted with orange circles. Coding sequences (CDSs) and untranslated regions (UTRs) are shown with green and yellow rectangles, respectively. Red and blue rectangles represent the kinase domains and the EF-hand motifs, respectively. The scale bars indicate gene and protein lengths.

Compared with representative domain architectures of CPKs or CCaMKs, the potential consequences of AS on domain architectures were categorized into nine types, namely truncated EF-hand motifs (Types 1–4), truncated kinase domain (Type 5), and remaining EF-hand motifs (Types 6–9) (Figure 5A). For CCaMKs, the representative domain architectures consisted of a kinase domain and three EF-hand motifs. We detected truncated protein variants of CCaMKs with an incomplete kinase domain (Type 5), as well as other CCaMK variants with only two or three EF-hand motifs but lacking the kinase domain (Types 7–8) (Figure 5B). CPK variants exhibited more diverse domain architecture types than CCaMK variants. Akin to CCaMK variants, we found similar architectures in CPK variants, i.e., variants with only EF-hand motifs (Types 6–7) and incomplete kinase domains (Type 5). Moreover, we identified variants containing a complete kinase domain with fewer EF-hand motifs (Types 1–3), as well as variants made up of only a complete kinase domain (Type 4) (Figure 5C). Although the functions of the truncated protein variants still need to be investigated, our analysis demonstrated that AS significantly contributes to the diversified domain architectures of CPKs and CCaMKs, enhancing the knowledge of AS-mediated Ca^2+^-dependent regulatory mechanisms of CPKs and CCaMKs.

### 2.7. Homoeolog Expression Patterns of CPKs and Related Kinases Across Tissues and Species

**Figure 6:**
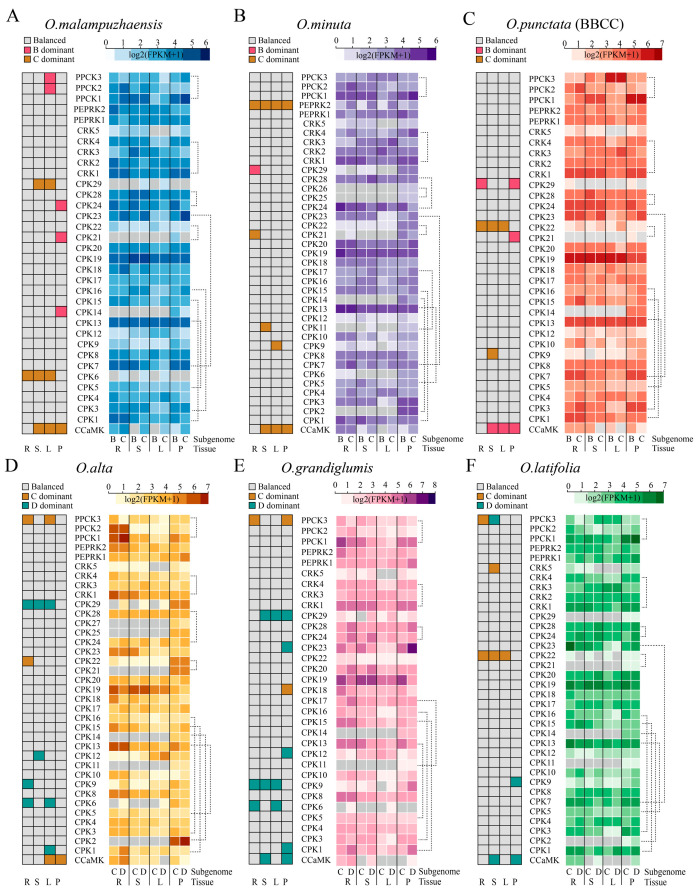
Homoeolog expression patterns of *CPKs* and related kinases across tissues and species. Homoeolog expression patterns were analyzed in two *Oryza* genome types, BBCC (**A**–**C**) and CCDD (**D**–**F**), in six allotetraploid species of wild rice: *O. malapuzhaensis* (**A**), *O. minuta* (**B**), *O. punctata* (**C**), *O. alta* (**D**), *O. grandiglumis* (**E**), and *O. latifolia* (**F**). Heatmaps on the left indicate the balanced expression pattern or a dominant expression towards a subgenome across roots (R), stems (S), leaves (L), and panicles (P). Gray indicates balanced expression between homoeologs. Red, brown, and cyan colors indicate preferential expression toward subgenomes BB, CC, and DD, respectively. The heatmaps on the right illustrate Log_2_ (FPKM+1)-normalized expression levels per homoeolog in root (R), stem (S), leaf (L), and panicle (P). Gray indicates that the gene was not expressed. The dashed lines link gene pairs.

In allopolyploid species, one homoeolog frequently has a dominant expression level over those of the others, and this is known as homoeolog expression bias (HEB). Understanding HEB will facilitate crop improvement and breeding programs by precisely modulating individual or multiple homoeologs [47]. Therefore, HEB patterns of the genes encoding CPKs and the related kinases were investigated in six allotetraploid species of wild rice accessions, comprising two genotypes: BBCC (*O. malampuzhaensis*, *O. minuta*, and *O. punctate* (BBCC)) and CCDD (*O. alta*, *O. grandiglumis*, and *Oryza latifolia*). Those that expressed genes with the 1:1 homoeologous relationship were included in the analysis. Most of the kinase-encoding genes, including *CPK3*, *PEPRK1*, and *PPCK1*, had balanced expression between subgenomes across tissues, demonstrating the stable HEB patterns of these genes. In contrast, we found dynamic HEB patterns between species and tissues. In the BBCC genome type, *CCaMK* copies from the subgenome CC were preferentially expressed in *O. malapuzhaensis* and *O. minuta*, while the BB subgenome copies were predominantly expressed in *O. punctate*. Similarly, in the CCDD genome type, *CCaMKs* in *O. alta* exhibited CC subgenome-biased expression in leaves and panicles, switching to balanced expression in roots and stems. The HEB patterns shifted in *O. grandiglumis* and *O. latifolia*, showing the DD subgenome-biased expressions in stems and panicles turned to balanced expression patterns in roots and leaves. *PEPRK2* exhibited an *O. minuta*-specific, biased expression pattern towards the CC subgenome copy across the investigated tissues, whereas the *PEPRK2* from other species of *Oryza* genus had balanced expression patterns, implying that HEB patterns are likely determined by the species rather than the subgenomes and are tissue-specific (Figure 6).

We observed similar HEB patterns between the duplicated gene pairs. For example, *CPK3*/*CPK16* and *CRK1*/*CRK4* in the selected genomes both showed balanced expression between the homoeologs across tissues, suggesting still-conserved expression patterns after gene duplication events. However, in some cases, the duplicated pairs exhibited diverse HEB patterns. *PPCK1*/*PPCK3* in *O. minuta* and *O. punctate* showed balanced expression across tissues, while distinct expression patterns were observed in the other species of *Oryza* genus. *CPK24*/*CPK28* in *O. malampuzhaensis* had disparate expression patterns in the panicle but balanced patterns in the root, stem, and leaf, confirming that HEB patterns of duplicated gene pairs shifted between genomes and tissues (Figure 6). Collectively, our HEB analysis provided insights into homoeolog expression patterns in the allotetraploid wild rice species, enhanced our knowledge of the allotetraploidization processes, and contributed to rice improvement.

### 2.8. Transcriptomal Atlas of CPKs and Their Related Kinases Underlines the Expression Dynamics and Indicates Potential Functions

**Figure 7:**
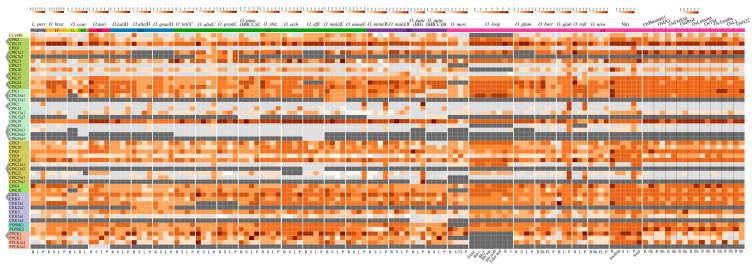
The expression patterns of the genes encoding CPKs and their related kinases in wild and cultivated rice. The expression levels of *CPKs* and their related kinases are visualized in the heatmap in log_10_ (FPKM). On the left of the heatmap, different colors highlight evolutionary groups of CPKs and their related kinases (from top to bottom, CCaMK, CPK subgroups I–IV, CRKs, PEPRKs, and PPCKs, respectively), while gray triangles connect duplicated gene pairs. Light gray indicates genes that were not expressed in tissues or species. Dark gray indicates orthologous genes absent in genomes/subgenomes. R, roots; L, leaves; S, stems; P, panicles; SAM, shoot apical meristem; Rhi, rhizomes; Sh, shoots.

To explore the expression dynamics and potential functions of genes encoding CPKs and related kinases, we complied multiple transcriptome datasets to form a comprehensive expression atlas in both wild and cultivated rice accessions. Firstly, we detected the expression patterns between the phylogenetic clades. CCaMKs were predominantly expressed in the roots of both wild and cultivated rice accessions, implying their conserved roles in root development or stress responses (Figure 7). However, there was no significant expression specificity among members within CPK subgroups, PPCKs, and PEPRKs, revealing the multi-faceted roles in different aspects of plant development.

Secondly, we compared the expression patterns of the duplicated gene pairs in *Oryza*. Our analysis demonstrated that the duplicated gene pairs exhibited different expression patterns. For example, for the gene pair *CPK5*/*CPK13*, expression levels of *CPK13* were generally higher than those of *CPK5* in all tested tissues and species, implying that *CPK5* and *CPK13* experienced a conservative evolutionary trajectory but diverse functions in *Oryza* (Figure 7). We observed asymmetric expression patterns in the *CPK11*/*CPK17* gene pair: *CPK17* exhibited dominant expression in most tested species, whereas *CPK11* was preferentially expressed in the panicles of *O. malampuzhaensis*, implying the subfunctionalization of *CPK11* in *O. malampuzhaensis*. For *CPK25*/*CPK26*, similar expression patterns for *CPK25*/*CPK26* were observed in panicles across species, indicating that they might function redundantly in panicle development.

Thirdly, we analyzed the expression patterns of species-specific duplicated gene pairs. *PPCK2a2* in *O. australiensis* and *CPK12a2* in *O. minuta* and *O. malampuzhaensis* were not expressed in the examined tissues, in contrast to their expressed homologs (*PPCK2a1* and *CPK12a1*), suggesting that they were undergoing pseudogenization (Figure 7). There was no significant expression difference between *CPK15a1* and *CPK15a2* in *O. malampuzhaensis*, indicating that they might function redundantly. *CPK29a2* in *O. punctata* (BB) had a higher expression level in the panicles than that of *CPK29a1*, implying neofunctionalization in *CPK29a2*. *CPK7a1* and *CPK7a2* in *O. alta* and *O. grandiglumis* were similarly expressed in the panicles, whereas biased expression was identified in the stem, showing that these elements could be functionally redundant in the panicles while playing divergent roles during stem development. Our analysis established a comprehensive expression atlas, exhibited the expression dynamics of CPKs and their related kinases across tissues and species, and characterized their potential functional roles, providing favorable genes encoding CPKs and their related kinases for rice improvement.

## 3. Discussion

The *Oryza* genus serves as a crucial reservoir of beneficial genes for rice improvement [10]. Several genes, such as *PROG1*, *Bph14*, and *SUB1A-1* from wild rice, have been identified and applied in rice breeding [48,49,50]. These examples highlight the value of species of *Oryza* genus, especially wild relatives, in addressing agricultural challenges. Here, with the rich genomic resources in *Oryza*, we selected the five evolutionarily close and functionally important kinases, CPKs and their related kinases, to apply an iGG strategy for genus-wide evolutionary analysis and favorable candidate gene mining. Previous studies in rice about *CPKs* and their related kinase gene families are limited in cultivated rice; however, *Oryza* species represent abundant favorable gene resources [28,29,30]. In this study, we identified 1217 *CPKs*, 212 *CRKs*, 123 *PPCKs*, 83 *PEPRKs*, and 41 *CCaMKs* in 34 *Oryza* genomes, representing almost all genome types and all three ecotypes in *Oryza* (Figure 1 and Figure 2 and Table S3). Our genus-wide analyses provided repertoires of *CPKs* and their related kinase gene families in *Oryza*, allowing us to investigate their evolutionary relationships, protein domain architectures, and expression dynamics, serving as a useful starting point for identifying *CPKs* and their related kinase gene families for rice improvement. It is greatly significant to investigate the five evolutionarily related and functionally important kinase family from a genus. Currently, population genomic data have been focused on two cultivated rice accessions and their ancestors (*O. rufipogon* and *O. barthii*), while there is a lack of data on the populations of the remaining wild relatives [11,51,52]. In the future, it will be worth exploring structural variations and single-nucleotide variations in gene family members considering population genomics.

The canonical CPKs and their related kinases were featured with distinct domain architectures. CCaMKs possessed a kinase domain, autoinhibitory domain, and a visinin-like domain containing three functional EF-hand motifs. The CPKs contained a variable N-terminal domain with the myristoylation and/or palmitoylation sites, followed by a serine/threonine kinase domain, an auto-inhibitory junction domain, and a CaMLD domain with four functional EF-hand motifs. CRKs shared similar architectures but with degenerated EF hands, which could not bind Ca^2+^. PPCKs and PEPRKs consisted only of a kinase domain, with differing C-termini [24,26]. In our analysis, most CPKs and their related kinases were in line with their canonical domain architectures. However, reduced numbers of functional EF-hand motifs, extended sequences in the C-terminus, and longer interspaced sequences between EF-hand motifs were observed in some CPKs (Figure 4). CPKs and CRKs have a common ancestor, originating from the fusion of the CaM-dependent protein kinase and CaM [25,27]. These variations in CPKs were possibly caused by fusion processes, recording *Oryza* evolution. The functions and Ca^2+^ affinities of these variations still need to be explored. According to our analysis, the domain architectures of duplicated gene pairs in CPKs and their related kinases were similar, suggesting the conservation of protein domain architectures in *Oryza* evolution. However, in the canonical CPKs, we identified the distinct arrangements of functional EF-hand motifs. CPKs in group I (CPK7 and CPK23) lacked EF-hand motifs in the N-lobe and C-lobe, while CPKs in group III (CPK3, CPK16, and CPK29) lost functional EF-hand motifs in the N-lobe of CaMLD (Figure 3). Considering the differential affinities in the N- and C-lobes of the CaMLD domain, these distinct arrangements of functional EF-hand motifs might have different consequences with two patterns toward calcium sensitivity. We speculated that the kinase activation levels of CPK7 and CPK23 might be affected at basal and elevated calcium concentrations, while the kinase activation levels of CPK3, CPK16, and CPK29 might not be affected at basal calcium concentrations but at elevated calcium concentrations. Our domain analysis revealed characteristic protein domain architectures in their related kinases and highlighted the diverse EF-hand motifs in CPKs and CCaMKs, contributing to heterogeneity in calcium sensitivity among CPKs and CCaMKs in *Oryza*.

Up to now, only limited articles have reported that the AS of CPKs and CCaMKs is involved in regulating protein domain architectures. No study has reported that CCaMKs are involved in AS-regulated protein domain architectures. The intron-retained *AtCPK28* spliced variant encodes a truncated protein lacking two high-affinity EF hands, and functions as a negative regulator in immune responses [53]. The AS-regulated truncated OsCPK17, lacking both the JD and CaM-like regulatory domain (similar to Type 4), loses Ca^2+^-binding capacity and in vitro kinase activity [54]. AtCPK25 is characterized by degenerated EF-hand motifs when compared with other AtCPKs (similar to Type 3), and displays Ca^2+^-independent kinase activity [55]. Collectively, these observations suggest that truncated CPKs with reduced functional EF-hand motifs in the CaM-like regulatory domain (Types 1–4) may partially or fully lose their ability to bind Ca^2+^. The variants of *OsCPK17* have been experimentally validated; however, so far, these cloned transcripts have not been completely curated in three Nipponbare reference genome annotations [56]. Therefore, it is possible that AS variants of *CPKs* and *CCaMKs* are not adequately curated. Thus, systematic efforts to improve transcript annotations are urgently required to explore the AS variants of *CPKs* and *CCaMKs*. Here, we utilized 427 RNA-seq libraries to analyze the effects of AS on protein domain architectures in *Oryza* and clarified the protein domain architectures of identified truncated protein variants, contributing to expanding our knowledge of the regulatory mechanisms of CPKs and CCaMKs (Figure 5). However, there are still some challenges to address: the large-scale identification of AS events and novel transcripts, the in vivo evidence for AS transcripts of *CPKs* and *CCaMKs*, and the modified functions of resulting truncated protein variants. Large-scale and high-throughput sequencing technologies, such as ISO-seq and Direct RNA sequencing, might be effective methods for identifying AS variants. Proteomics data mining and function studies might be the solutions for the veracity and modified functions of AS variants.

Previous studies have reported expression patterns of *OsCPKs* and *OsCRKs* in cultivated rice, and their results showed that *OsCPKs* and *OsCRKs* are expressed broadly but in a tissue-specific manner [28,30,57,58,59]. In our analysis, *CCaMK* displayed dominant expression in roots among wild rice and cultivated rice, while no apparent tissue-specific expression patterns among other groups were observed. Regarding the expression of duplicated gene pairs, our analysis results are supported by previous reports [28,59], which also found similar expression levels between gene pairs *CPK25*/*CPK26* and preferential expression towards *CPK13* compared with *CPK5*. We further compared the expression patterns of species-specific duplicated gene pairs to infer their potential functional conservation and divergence, e.g., *CPK15a1/CPK15a2* were similarly expressed in *O. malampuzhaensis*, indicating their redundant functions. The altered expression patterns of *CPK29a2* in *O. punctata* (BB) suggested their functional divergence (Figure 7). As reported, *CPKs* and their related kinase genes respond to biotic and abiotic stresses and phytohormones, highlighting the shortcomings of our study. Due to limited transcriptome data on wild rice and weedy rice, no public stress- and phytohormone-treated transcriptome data were included in our analysis. Our expression profile analysis exhibited the transcriptome landscapes of genes encoding CPKs and their related kinases in *Oryza*, emphasizing the expression patterns of duplicated gene pairs from the perspective of the genus.

## 4. Materials and Methods

### 4.1. Gene Identification of CPKs and Their Related Kinases

We conducted this study to comprehensively identify genes encoding CPKs and their related kinases, CRKs, PEPRKs, PPCKs, and CCaMKs, in *Oryza*. The 34 *Oryz* genome assemblies, including 20 wild rice, 12 cultivated rice, and 2 weedy rice accessions, along with *L. perrieri* as an outgroup, were selected for further analysis. The detailed information of the above-mentioned genome assemblies is provided in Table S1. To identify genes encoding CPKs and their related kinases in *Oryza*, we initially re-searched these gene families via Bitacora v1.4 [42] using the full mode against three Nipponbare reference annotations, including MUS [39], AGIS-1.0 [40], and RAP-DB [41]. Then, the protein sequences of CPKs and their related kinases from the MUS annotation and *Arabidopsis* were used as seeds for BLASTP v2.16.0+ and HMMER v3.4 searches with the protein kinase domain (PF00069) and Ca^2+^-binding domain (PF13499 and PF13833). The proteins searched via BLASTP and HMMER, together with orthologous and paralogous proteins identified via MCScanX [60] and GeneTribe v1.2.1 [61], were used as putative candidates. The protein domains of putative candidates were validated via local InterProScan [62] (version: 5.71–102.0) and ScanProsite [63] (Release version: 5-February-2025). The confirmed proteins were categorized into *CCaMK*, *CPK*, *CRK*, *PEPRK*, and *PPCK* gene families based on protein similarity matrix and their contained domains.

### 4.2. Construction of Phylogenetic Tree

For the construction of a phylogenetic tree, the protein sequences of CPKs and their related kinases in *Oryza* and *Arabidopsis* were aligned using MUSCLE v3.8.31 [64] with default parameters. After alignment trimming via trimAl v1.5 [65], the phylogenetic tree was inferred via Maximum Likelihood implemented in RaxML v8.2.12 [66] using the PROTGAMMAJTT algorithms with 1000 bootstrap replicates.

For constructing the species tree, a coalescent-based species tree approach was employed to infer species phylogeny. Allotetraploid wild rice accessions were divided based on their genomic compositions. Regarding *O. coarctata*, its genome was aligned using nucmer v4.0.0beta2 [67] against OcoaRS1 (GCA_036417745.1), a non-public genome assembly version. Meanwhile, subgenomes of *O. coarctata* were phased via SubPhaser v1.2.6 [68]. Orthofinder v2.5.4 [69] was employed to infer orthologous genes based on the longest transcripts. The 2297 single-copy orthologous genes were aligned using MUSCLE v3.8.31 [64]. Subsequently, the gene trees were constructed using RaxML v8.2.12 [66] with 100 bootstrap replicates using the PROTGAMMAJTT model. The coalescent-based species tree was estimated via ASTRAL v5.7.8 [70] based on constructed gene trees, with *L. perrieri* serving as the outgroup. The phylogenetic trees were visualized and annotated using Interactive Tree of Life (https://itol.embl.de/ (accessed on 21 February 2025)) [71] for graphical optimization and topological annotation.

### 4.3. Gene Structure and Protein Domain Analysis

For gene structure analysis, gene features were extracted from genomics data and reconstructed transcripts and visualized using TBtools-II v2.225 [72]. The protein domain architectures of identified CPKs and their related kinases were scanned with ScanProsite. Profiles PS50011 and PS50222 were regarded as a protein kinase domain and an EF-hand motif, respectively. Pattern PS00018 was considered a Ca^2+^-binding site, namely the functional EF-hand motifs.

Myristoylation and palmitoylation sites were predicted using GPS-Lipid [73]. Protein parameters were computed with ProtParam in Expasy (https://web.expasy.org/protparam/ (accessed on 25 February 2025)). Subcellular localizations of CPKs and their related kinases were predicted using DeepLoc (https://services.healthtech.dtu.dk/services/DeepLoc-2.0/ (accessed on 23 March 2025)) [74].

### 4.4. Gene Duplication and Synteny Analysis

All-versus-all BLASTP (the E-value cutoff of 1 × 10^−10^) between genomes and subgenomes were first performed. Subsequently, the collinearity and homology between inter-genomes were inferred with GeneTribe v1.2.1 [61]. The collinearity blocks between intra-genomes were scanned via MCScanX [60]. Gene duplication types were categorized into WGD, TD, PD, TRD, and DSD using the DupGen finder pipeline [75].

### 4.5. Reference-Based Transcriptome Assembly and Expression Quantification

First, Raw RNA-seq reads were processed using fastp v0.23.4 [76] to trim low-quality bases and remove adapters. Then, the clean reads were mapped to the corresponding genomes with HISAT2 v2.1.0 [77], with the intron length ranging from 50 to 5000 bp. Followed by transcript assembly for each library performed with StringTie v2.2.3 [78] and Scallop v0.10.5 [79], the merge mode implemented in StringTie v2.2.3 was used to integrate reconstructed transcripts in all samples and generate nonredundant transcripts. Subsequently, novel transcripts were filtered using GffCompare v0.12.6 [80], retaining those with class codes (k, m, n, x, i, and o). The protein-coding potentials of the novel transcripts were predicted via CPC2 v0.1 [81]. Finally, TransDecoder v5.7.1 (Haas, BJ. https://github.com/TransDecoder/TransDecoder (accessed on 25 November 2024)) was used to identify the candidate coding regions of novel coding transcripts via combination with the search for Pfam using hmmscan and BLASTP results against the local protein database, including SwissProt, Uniprot, and protein sequences from reference genomes and pan-genomes of rice [12,13,17,52], *Arabidopsis* [82], barley [83], and wheat [84]. To analyze the effects of AS events on protein domain architectures, the reconstructed transcripts of the identified genes encoding CPKs and CCaMKs were selected, and combined with transcripts in genome annotations. Subsequently, their corresponding protein sequences were used for protein domain analysis.

For gene expression quantification, StringTie v2.2.3 [78] was utilized to estimate the fragments per kilobase of transcript per million mapped reads (FPKMs) as the gene expression levels. Expression profiles of Nipponbare and *O. glaberrima* were obtained from PPRD (https://plantrnadb.com/ricerna/ (accessed on 28 February 2025)) with accessions (PRJNA482217 and PRJNA13765) [85]. Homoeolog expression bias (HEB) was performed according to the previous method [47]. We included expressed homologs (FPKM > 0.5) with the 1:1 homoeologous relationship between subgenomes, calculated the relative contributions of each subgenome per homeolog, and assigned the homoeolog expression bias category based on their Euclidean distances from observed normalized expression to three ideal categories. The ideal normalized expression bias patterns for the three categories are provided in Table S6.

## 5. Conclusions

In summary, we applied an iGG strategy for genus-wide evolutionary analysis of the five evolutionarily close and functionally important kinases, namely *CPKs* and their related kinases, and provided several valuable findings regarding CPKs and their related kinases. (1) Through *Oryza*-wide identification and analysis, we provide comprehensive repertoires of *CPKs* and their related kinases in 34 *Oryza* genomes for evolutionary relationships, protein domain architectures, and expression analysis. (2) We elucidate distinct gene duplication types contributing to the expansion of genes encoding *CPKs* and their related kinases in wild rice by establishing a colinear map in *Oryza*. (3) Protein domain architecture analysis reveals the divergent EF-hand motifs of CPKs and CCaMKs in *Oryza*, and we find that alternative splicing regulates the domain architecture rearrangement of CPKs and CCaMKs, deepening our knowledge of their regulation mechanisms. (4) Transcriptome landscapes of genes encoding CPKs and their related kinases exhibit their expression dynamics and potential functions in *Oryza*, emphasizing divergent homoeolog expression patterns between species and tissues. In future studies, our findings from analyzing protein domain architectures will undergo experimental investigations to elucidate regulation mechanisms, and mining gene resources from wild relatives will be applied to other grass or plant species.

## Data Availability

The data presented in this study are available in the article and the Supplementary Materials.

## Supplementary Materials

The following supporting information can be downloaded at: https://www.mdpi.com/article/10.3390/plants14101542/s1, Figure S1: Re-identification of genes encoding CPKs and their related kinases in three Nipponbare reference genomes; Figure S2: Conservative *CCaMK* locus in *Oryza*; Figure S3: Expansion of *CPK12* in *Oryza*; Figure S4: Divergent protein domain architectures encoded by duplicates of *CPK12* gene pairs; Figure S5: Conserved domain architectures between PPCK2 copies mediated by transposed duplication; Figure S6: Conserved domain architectures encoded by WGD-driven *CPK7* gene pairs; Figure S7: Conserved domain architectures encoded by *CPK15* gene pairs mediated by proximal duplication; Figure S8: Homoeolog expression patterns of *CPKs* and their related kinases across species and tissues; Figure S9: Revised annotation of *OaltaCPK26a1*; Table S1: Meta information of 35 genome assemblies used in this study. Table S2: Information of the identified CPKs and their related kinases in three Nipponbare reference genomes. Table S3: Information of the identified CPKs and their related kinases in the present study. Table S4: RNA-seq datasets used in this study. Table S5: Information of reconstructed transcripts of genes encoding CPKs and their related kinases. Table S6: Definition of homoeolog expression bias categories.

## Abbreviations

The following abbreviations are used in this manuscript:

CPKs	Calcium-dependent protein kinases
CRKs	CPK-related kinases
PPCKs	Phosphoenolpyruvate carboxylase kinases
PEPRKs	PPCK-related kinases
CCaMKs	Calcium and calmodulin-dependent kinases
VNTD	Variable N-terminal domain
JD	Junction domain
CaM	Calmodulin
CaMLD	Calmodulin (CaM)-like domain
CNV	Copy number variation
WGD	Whole-genome duplication
TD	Tandem duplication
PD	Proximal duplication
TRD	Transposed duplication
DSD	Dispersed duplication
AS	Alternative splicing
HEB	Homoeolog expression bias
