# Supplementary material for: Genus-Wide Pan-Genome Analysis of Oryza Calcium-Dependent Protein Kinase Genes and Their Related Kinases Highlights the Complexity of Protein Domain Architectures and Expression Dynamics
Source: Plants (Basel). 2025 May 20;14(10):1542. doi: 10.3390/plants14101542 (PMC12115312; doi:10.3390/plants14101542)
Supplement: Supplementary file 1 [file plants-14-01542-s001.zip › plants-3587130-supplementary/Supplementary Figures and Table S6.pdf]

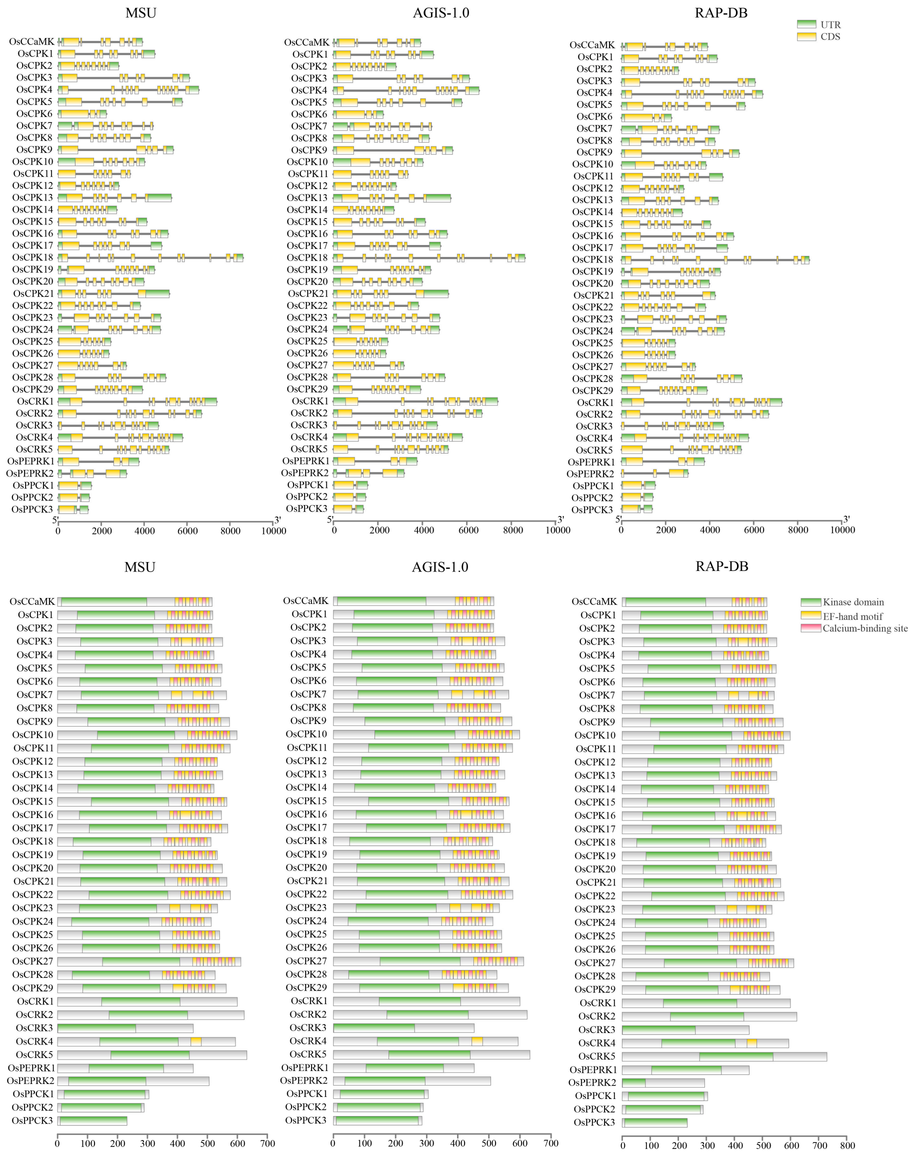

Figure S1. Re-identification of genes encoding CPKs and their related kinases in three Nipponbare reference genomes. Our re-identification analysis of genes encoding CPKs and their related kinases were conducted in three reference Nipponbare genomes, including MUS, AGIS-1.0, and RAP-DB. (**A**) Exon-intron structures of canonical genes encoding CPKs and their related kinases from the three annotations. (**B**) Comparison of protein domain structures among CPKs and their related kinases from the three annotations. Kinase domains, EF-hand motifs, and calcium-binding sites are shown with green, yellow, and red rectangles, respectively. The scale bars represent the gene or protein lengths.

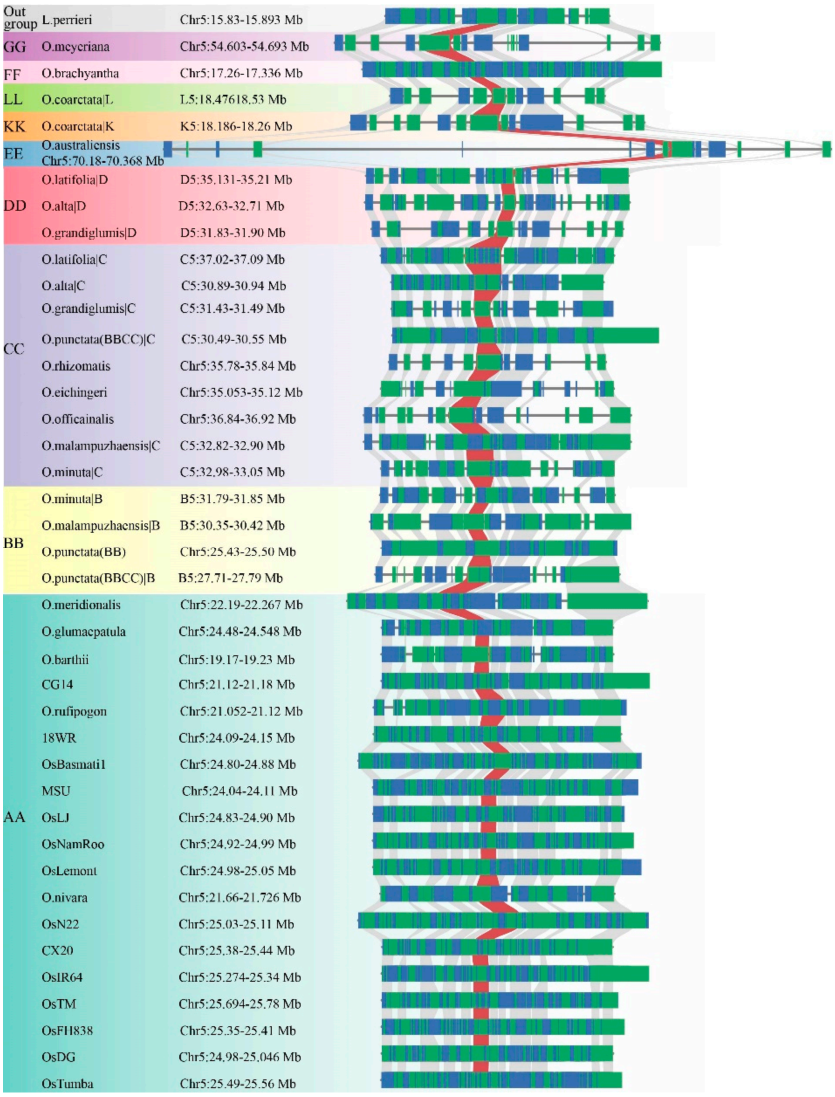

Figure S2. Conservative *CCaMK* locus in *Oryza*. Inter-genome/subgenome syntenic analysis of the *CCaMK* locus in 41 genomes/subgenomes revealed conservative evolutionary history of *CCaMK* in *Oryza*. The red line highlights inter-genome syntenies of *CCaMK* in 41 genomes/subgenomes with nine distinct genome types (AA, BB, CC, DD, EE, KK, LL, FF, and GG). Chromosomal positions of *CCaMK* locus are indicated.

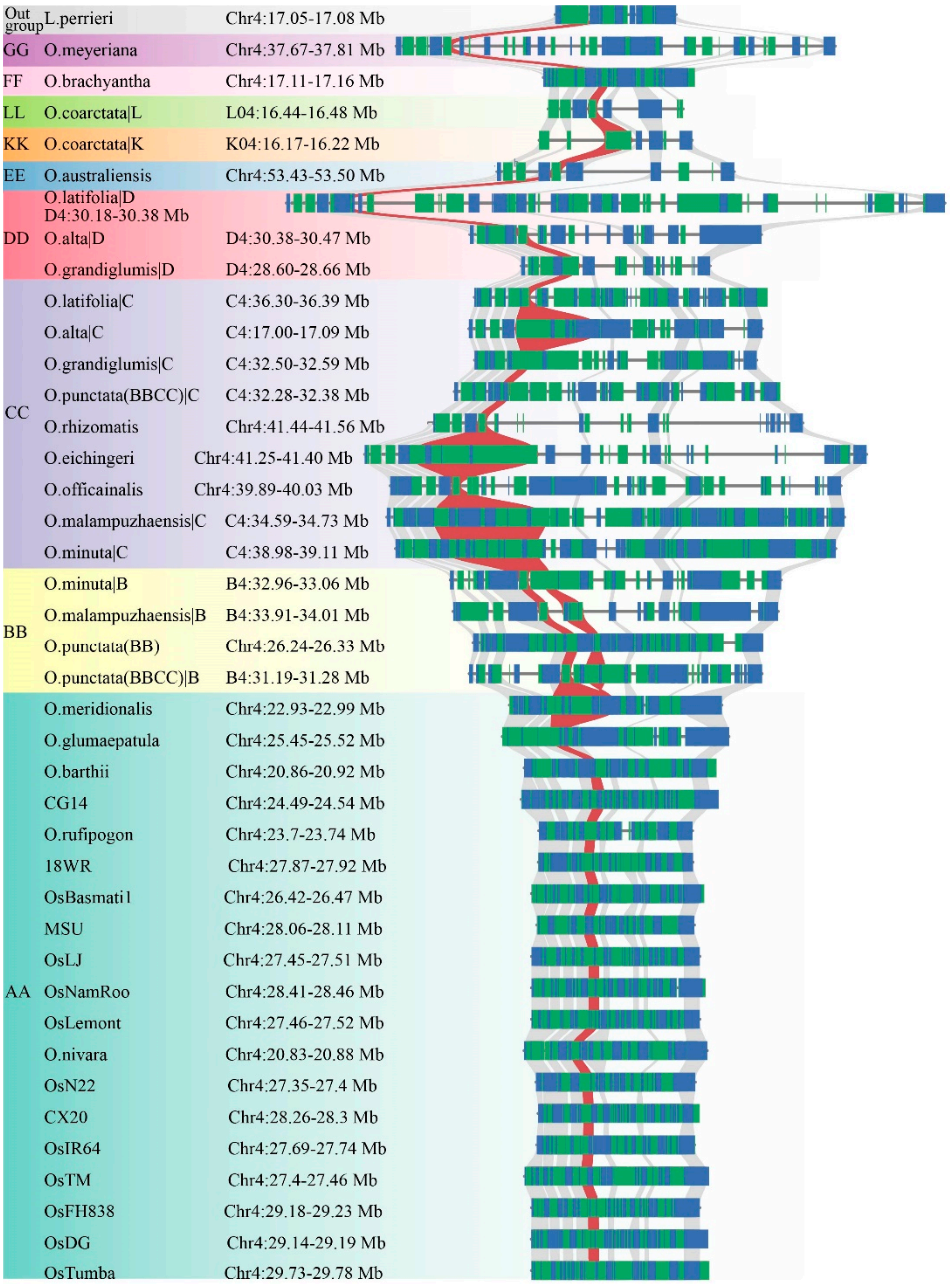

Figure S3. Expansion of *CPK12* in *Oryza*. Inter-genome/subgenome syntenic analysis of *CPK12* locus in *Oryza* revealed subgenome-specific expansions in BB. The red line highlights inter-genome syntenies of *CPK12* in 41 genomes/subgenomes with nine distinct genome types (AA, BB, CC, DD, EE, KK, LL, FF, and GG). Chromosomal positions of *CPK12* locus are indicated.

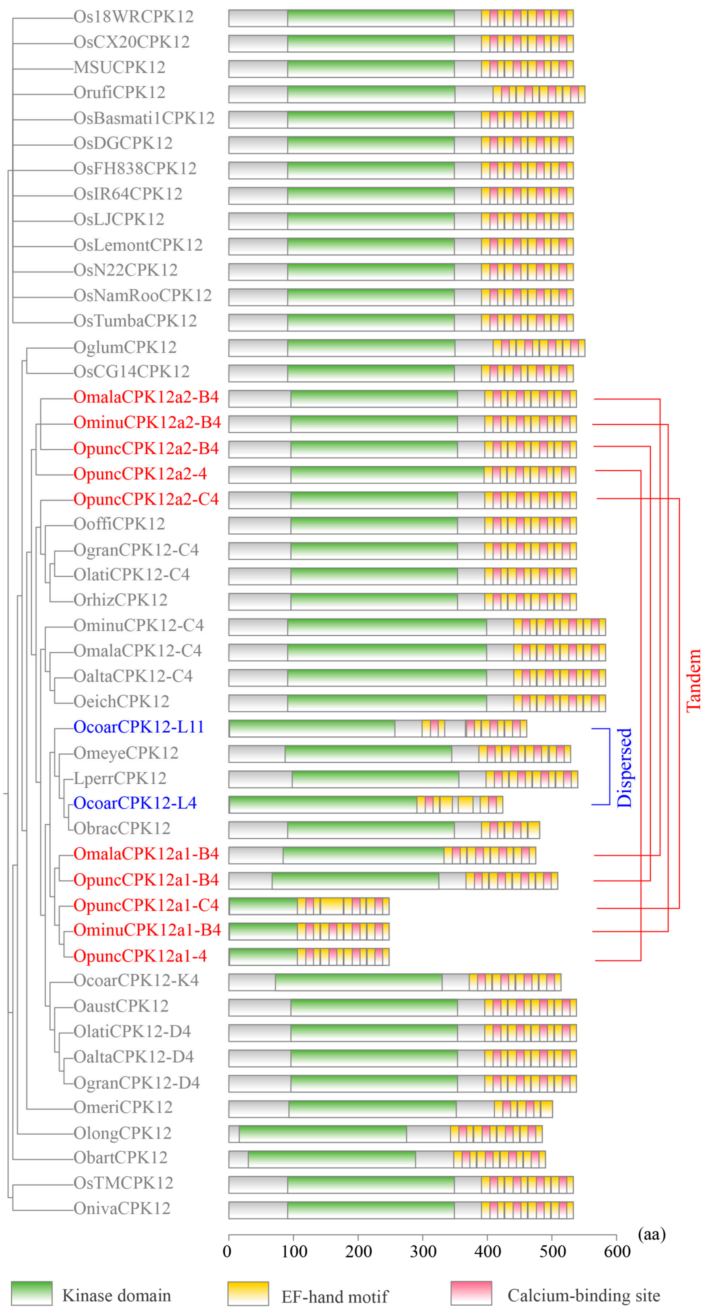

Figure S4. Divergent protein domain architectures encoded by duplicates *CPK12* gene pairs. *OcoarCPK12-L4/OcoarCPK12-L11* was identified as dispersed duplication pairs. *OmalaCPK12a2-B4/OmalaCPK12a1-B4*, *OminuCPK12a2-B4/OminuCPK12a1-B4*, *OpuncCPK12a2-4/OpuncCPK12a1-4*, *OpuncCPK12a2-C4/OpuncCPK12a1-C4*, and *OpuncCPK12a2-B4/OpuncCPK12a1-B4* were identified as tandem duplication gene pairs. Their protein domain architectures become divergent. Kinase domains, EF-hand motifs, and calcium-binding sites are shown with green, yellow, and red rectangles, respectively. The scale bar represents protein length.

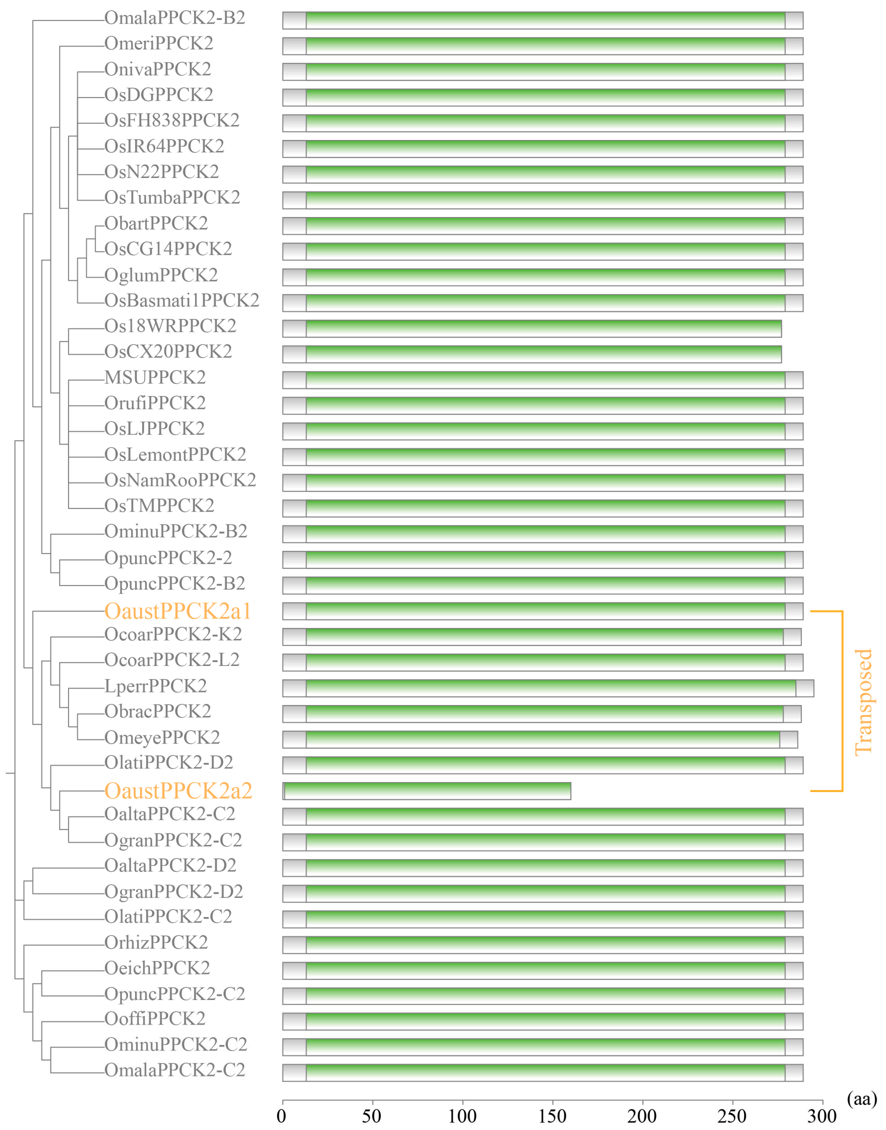

Figure S5. Conserved domain architectures between PPCK2 copies mediated by transposed duplication Transposed duplication drove *PPCK2* expansion into two copies, while protein domain architecture was altered in OaustPPCK2a2, with truncated kinase domain. The green rectangles represent the kinase domains. The scale bar represents protein length.

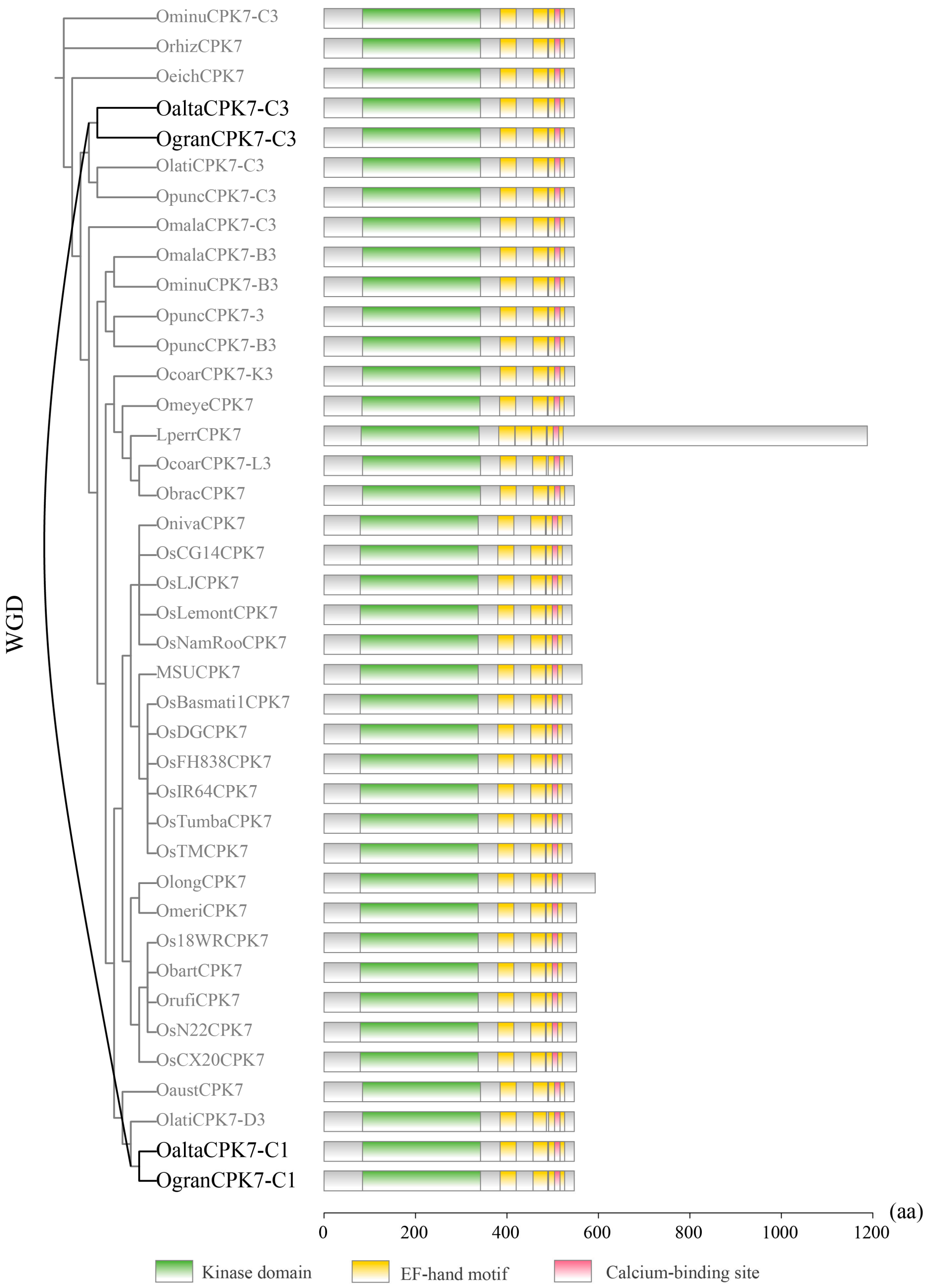

Figure S6. Conserved domain architectures encoded by WGD-driven *CPK7* gene pairs *OaltaCPK7-C1*/*OaltaCPK7-C3* and *OgranCPK7-C1*/*OgranCPK7-C3* were duplicated by whole genome duplication (WGD), respectively. The two copies both retained the identical protein domain architecture. Kinase domains, EF-hand motifs, and calcium-binding sites are shown with green, yellow, and red rectangles, respectively. The scale bar represents protein length.

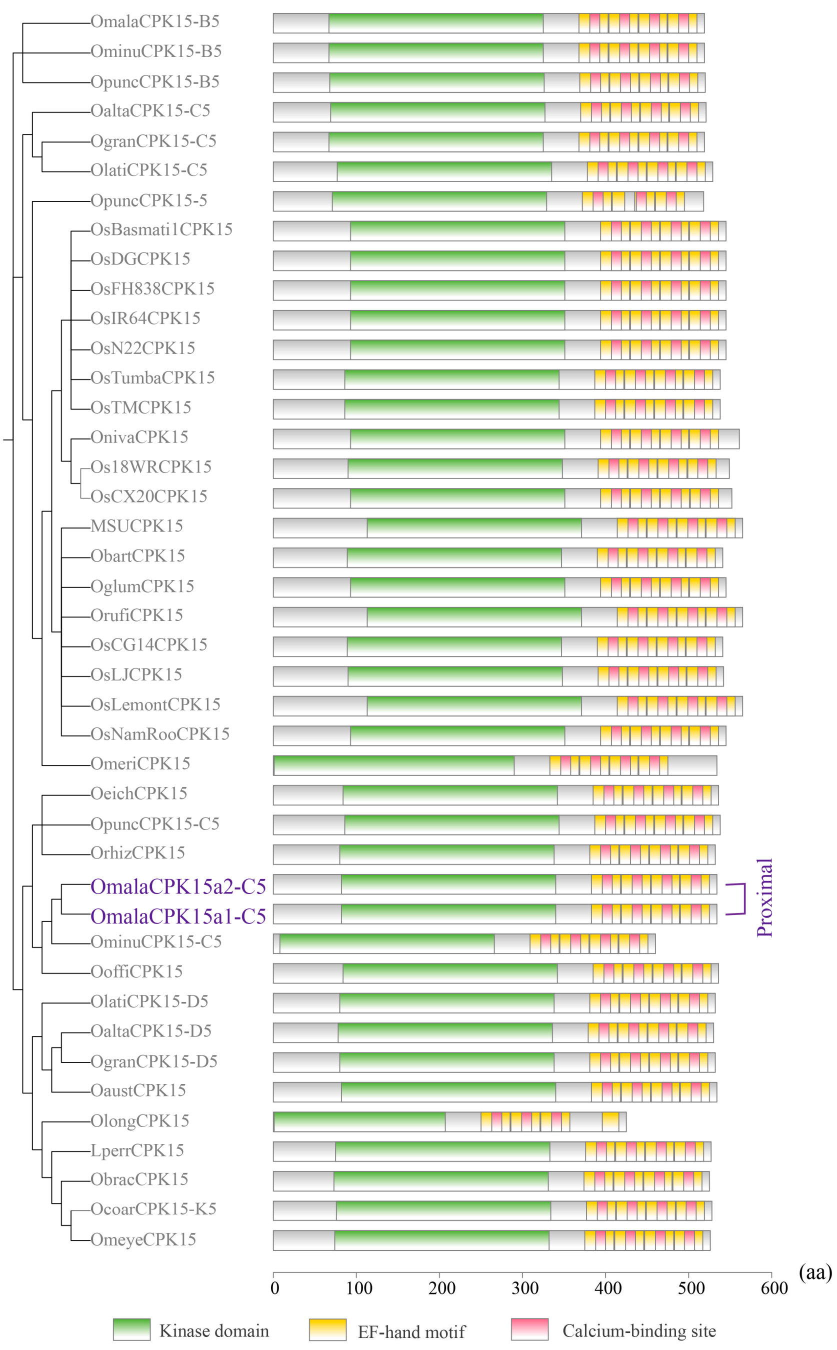

Figure S7. Conserved domain architectures encoded by *CPK15* gene pairs mediated by proximal duplication. *CPK15* in *O. malampuzhaensis* underwent proximal duplication with two copies, yet maintained the same domain architecture. Kinase domains, EF-hand motifs, and calcium-binding sites are shown with green, yellow, and red rectangles, respectively. The scale bar represents protein length.

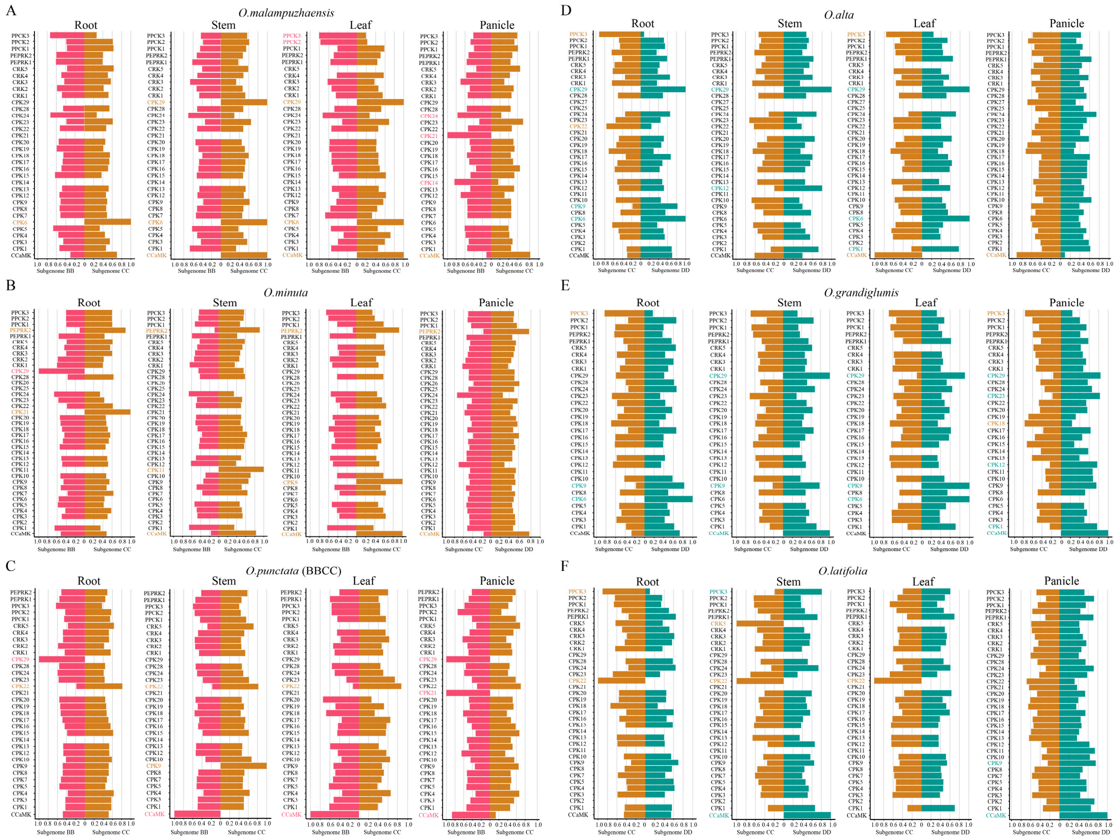

Figure S8. Homoeolog expression patterns of *CPKs* and their related kinases across species and tissues. Homoeolog expression pattern analysis of *CPKs* and their related kinases were conducted with two genome types, BBCC (**A-C**) and CCDD (**D-F**), in six allotetraploid species of wild rice, including *O. malapuzhaensis* (**A**), *O. minuta* (**B**), *O. punctata* (**C**), *O.alta* (**D**), *O. grandiglumis* (**E**), and *O.latifolia* (**F**). The relative expression contribution of subgenome homoeologs were calculated and visualized in bidirectional bar charts. Red, brown, and cyan colors mean preferential expression towards subgenome BB, CC, and DD, respectively. Gene names in black mean the balanced homoeolog expression pattern.

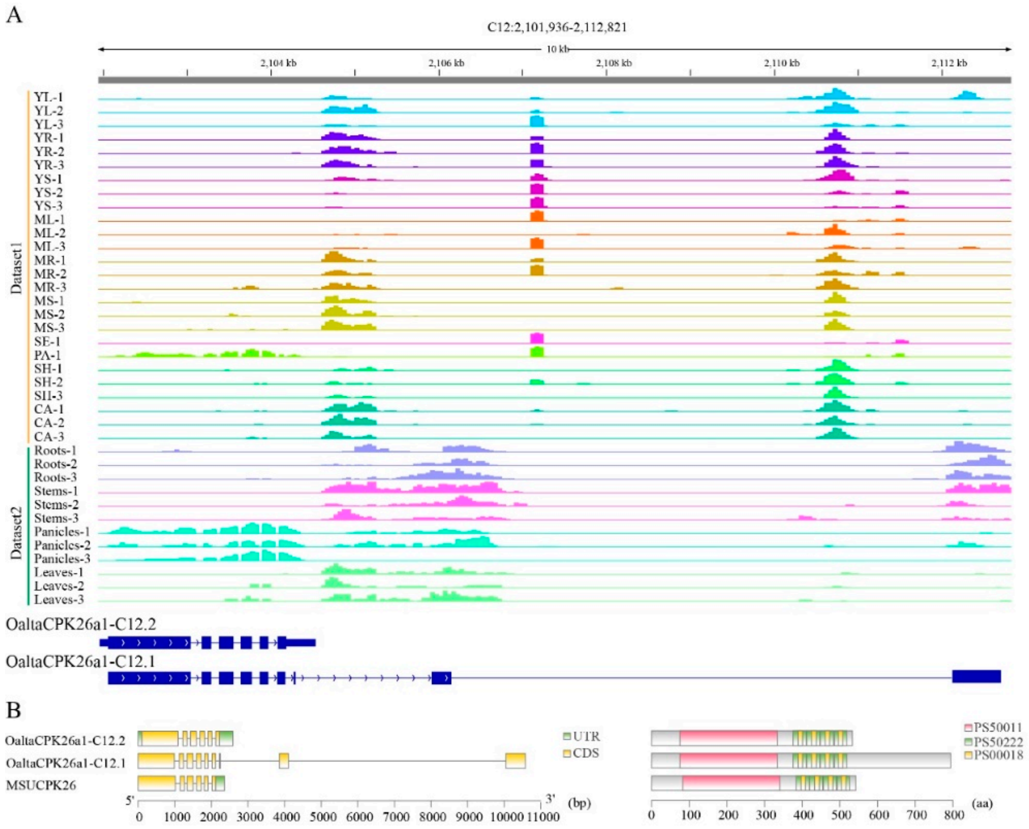

Figure S9. Revised annotation of *OaltaCPK26a1*. (A) Visualization of mapped reads of *OaltaCPK26a1* using two RNA-seq datasets. *OaltaCPK26a1-C12.2* was the assembled transcript in our RNA-seq analysis, while *OaltaCPK26a1-C12.1 was the* transcript provided in the annotation. (B) Exon–intron structures and protein domains of OaltaCPK26a1-C12.1, OaltaCPK26a1-C12.2, and MSUCPK26, orthologous gene in Nipponbare reference genome. Coding sequences (CDS) and untranslated regions (UTR) are shown with green and yellow rectangles, respectively. The scale bars indicate gene and protein lengths.

Table S6 Definition of homoeolog expression bias categories. Genotype BBCC is as an example for illustration. BB and CC represent the relative expression levels of the BB and CC subgenome homoeologs, respectively.

Category	BB	CC
Balanced	0.5	0.5
B dominant	1	0
C dominant	0	1
